# Anomaly detection and removal strategies for in-line permittivity sensor signal used in bioprocesses

**DOI:** 10.3389/fbioe.2025.1609369

**Published:** 2025-07-30

**Authors:** Emils Bolmanis, Selina Uhlendorff, Miriam Pein-Hackelbusch, Vytautas Galvanauskas, Oskars Grigs

**Affiliations:** ^1^ Laboratory of Bioengineering, Latvian State Institute of Wood Chemistry, Riga, Latvia; ^2^ K. Tars Lab, Latvian Biomedical Research and Study Centre, Riga, Latvia; ^3^ Institute of Biomaterials and Bioengineering, Riga Technical University, Riga, Latvia; ^4^ Institute for Life Science Technologies ILT.NRW, OWL University of Applied Sciences and Arts, Lemgo, Germany; ^5^ Department of Automation, Kaunas University of Technology, Kaunas, Lithuania

**Keywords:** in-situ, permittivity, dielectric spectroscopy, signal preprocessing, dynamic threshold, static threshold, anomaly validation, *Pichia pastoris*

## Abstract

**Introduction:**

In-line sensors, which are crucial for real-time (bio-) process monitoring, can suffer from anomalies. These signal spikes and shifts compromise process control. Due to the dynamic and non-stationary nature of bioprocess signals, addressing these issues requires specialized preprocessing. However, existing anomaly detection methods often fail for real-time applications.

**Methods:**

This study addresses a common yet critical issue: developing a robust and easy-to-implement algorithm for real-time anomaly detection and removal for in-line permittivity sensor measurement. Recombinant *Pichia pastoris* cultivations served as a case study. Trivial approaches, such as moving average filtering, do not adequately capture the complexity of the problem. However, our method provides a structured solution through three consecutive steps: 1) Signal preprocessing to reduce noise and eliminate context dependency; 2) Anomaly detection using threshold-based identification; 3) Validation and removal of identified anomalies.

**Results and discussion:**

We demonstrate that our approach effectively detects and removes anomalies by compensating signal shift value, while remaining computationally efficient and practical for real-time use. It achieves an F1-score of 0.79 with a static threshold of 1.06 pF/cm and a double rolling aggregate transformer using window sizes *w1* = 1 and *w2* = 15. This flexible and scalable algorithm has the potential to bridge a crucial gap in process real-time analytics and control.

## 1 Introduction

The quest for efficiency, safety and sustainability is driving new developments in the bioprocess industry. This includes monitoring, controlling and predicting cell cultivation processes as continuously as possible and in real-time. Achieving this requires comprehensive process knowledge as well as the analysis of various process parameters, which are recorded using modern sensor technology (Mandenius and Gustavsson, 2015). In-line sensors, which do not influence the process or the product and continuously supply process data in real-time, are particularly important here. They can ensure early detection of deviations, such as nutrient limitations, and are thus able to optimally determine feeding profiles or harvesting times, for example. Since a single sensor signal can rarely provide information about such a complex process as cell cultivation, it is worthwhile to use mathematical models to fuse the signals of different sensors into a so-called soft sensor. In the development of such soft sensors, the quality of the data is of crucial importance so that the mathematical model to be created on the basis of the data is not negatively influenced (Warne et al., 2004; Brunner et al., 2021).

According to the International Standard ISO/IEC 25012:2008, data quality is defined as ‘degree to which the characteristics of data satisfy stated and implied needs when used under specified conditions’, where data quality characteristics are defined as ‘category of data quality attributes that bears on data quality’ (International Organization for Standardization, 2008). For a detailed list of the 15 characteristics defined there, we refer to this standard. Among other important characteristics, the most important one to consider is credibility. Credibility refers to the extent to which data possesses attributes that are considered authentic and trustworthy by users within a given context of application (International Organization for Standardization, 2008). At this point, the aforementioned process knowledge comes into play, helping to assess whether or not the captured sensor data is true and believable.

An example for this is the recorded trend of the viable biomass (more precisely, the signal which is correlated with it), which, depending on the process control strategy, should correspond to classical growth kinetics. If irregularities such as spikes or signal shifts are detected, this indicates in most cases an anomaly of the sensor signal and not of the true viable biomass value. To record such a signal in-line and in real-time, permittivity probes that can infer viable cell density are suitable, for example. These probes polarize cells with intact cell membranes through an alternating electric field, while dead cells with damaged membranes are not polarized and thus not measured (Metze et al., 2020). However, it is important to correlate the probe signals with off-line reference analytics to make a qualitative statement about the viable cell density (Ramm et al., 2023).

Depending on the sensor and the underlying measurement technology, signal anomalies can be caused by external process changes, such as a change in agitator speed, the addition of an antifoam agent (Grigs et al., 2021a) or movement of bubbles near the sensor tip (Fehrenbach et al., 1992; Konstantinov et al., 1992; Münzberg et al., 2017; Katla et al., 2019; Brignoli et al., 2020). In such cases, the recorded sensor signal does not reflect the true viable biomass value. If this erroneous, unreliable data were fed into a mathematical model without any preprocessing, this would lead to a flawed model. Therefore, it is of utmost importance to detect and filter out signal anomalies in the preprocessing step (Kadlec et al., 2009; Jiang et al., 2021).

However, there are both process-inherent and application-dependent aspects that must be considered in such a preprocessing task. One of the process-inherent aspects is that the recorded viable cell density signal is time dependent, as bioprocesses generally are, which is why the signal is non-stationary. This means that changes in the mean (increasing signal due to increasing cell biomass) and variance (e.g., increased signal noise at low cell densities) can be observed. Another obvious but important aspect to consider is the fact that both data preprocessing and modeling must be possible in real-time, i.e., during the ongoing process. The acceptable latency between the time when an event occurs in the process and the time it is detected depends on the application’s goal. If the intended use of the developed soft sensor is solely for monitoring purposes, for example, there are lower demands placed on latency compared to when it is intended for control, where rapid responsiveness is of great importance. Dependent on this, filters and algorithms are used in data preprocessing and mathematical model building, which may entail a time delay or high computational power. Further requirements for streaming algorithms can be found in (Ahmad et al., 2017; Blázquez-García et al., 2022).

The existing anomaly detection techniques can be categorized based on input dimensionality, learning type category, and method family. Input dimensionality differentiates between univariate and multivariate data types and describes the extent to which algorithms can handle inter-variable. In terms of learning types, techniques can be classified as unsupervised, semi-supervised, or supervised. The method families can be broadly divided into six categories: forecasting, reconstruction, distance, encoding, distribution, and tree methods (Schmidl et al., 2022). However, none of these categorizations provide insight into whether the respective algorithms are fundamentally suitable for real-time application in non-stationary processes.

Regarding the categorization of different anomalies, a common distinction is made between point anomalies and sequential anomalies (Schmidl et al., 2022), with the former often appearing as contextual anomalies in time series data (Chandola et al., 2009). This is because a signal value recorded at a specific time point may represent an anomaly due to its context but would not be classified as an anomaly if it occurred at a different time. Since this context dependency complicates anomaly detection, it is beneficial to transform the sensor signal data in such a way that the contextual information is removed, leaving point anomalies without context dependency. For detecting those, Chandola et al. propose classifying anomaly detection techniques into six categories, including, for example, classification based techniques, nearest neighbor based techniques, clustering based techniques and statistical techniques (parametric methods such as gaussian model-based and non-parametric methods such as histogram-based) (Chandola et al., 2009).

Both, traditional, manual anomaly detection and modern machine learning methods have the disadvantage of rarely working in real-time (Hill and Minsker, 2010; Ahmad et al., 2017). Even algorithms that could theoretically be applied in real-time do not inherently guarantee that their implementation as a streaming algorithm will work in practice. In this regard, the data acquisition rate and the computation time of the algorithm must always be taken into account, which, depending on the process, may make real-time integration impossible (Blázquez-García et al., 2022).

Assuming that our transformed, context-free data follows a Gaussian distribution, we have chosen Gaussian Model-Based techniques for this study. These methods offer the advantage that they can be applied as streaming algorithms due to their low computational power and are also relatively easy to understand and implement. The latter was important to us so that a broad readership can apply our algorithm to their own sensor signal data.

To the best of our knowledge, the topic of anomaly detection and removal in sensor signal data for recording in-line viable cell biomass in bioprocesses remains largely unexplored in the existing literature. The only known contribution in this area is the work by Grigs et al. (2021a), which forms the basis of the present study. Building on this groundwork, our study addresses a critical gap and pioneers further exploration into this underdeveloped yet essential field.

Using permittivity measurements from recombinant *P. pastoris* fermentations, this study aimed to develop an algorithm for detecting and removing signal anomalies in real-time. To achieve this goal, three main questions needed to be addressed.

1. How to overcome the non-stationarity of the signal?
2. How to detect anomalies?
3. How to remove anomalies?

Based on the three questions above, our approach can be divided into three consecutive steps, into which both, this study and the algorithm, are divided. Step 1) is the signal preprocessing, which includes the reduction of noise and the transformation of the smoothed signal to remove context dependency. Step 2) is the anomaly detection and the associated selection of an appropriate threshold, based on which an anomaly is classified as such. Step 3) is the validation of anomalies and their removal.

Our requirements for the algorithm included the possibility of real-time in-line application and minimal complexity in terms of mathematical and computational aspects.

## 2 Theory

### 2.1 Signal preprocessing

With regard to the variance of the signal over time, it becomes apparent that data smoothing is necessary to reduce noise. To minimize signal noise before the actual signal anomaly detection and removal, various smoothing methods seem suitable, which will be discussed in more detail below. For all methods, the window size *w* is a freely selectable and optimizable parameter. However, it should be noted that this choice involves a trade-off when implementing the filter in real-time: the larger the chosen window size *w*, the stronger the noise reduction, but also the greater the time delay between input (raw signal) and output (smoothed signal), which can be described by (*w*-1)/2 (Harju et al., 1996).

The moving mean smooths signals by calculating the mean of data points over a specific window size, which is typically centered on the point being analyzed. The moving median works the same way, except that the median is used as the aggregation function instead of the mean.

In the Gaussian filter, a weight is calculated for each data point within the selected window based on an underlying Gaussian function, and the value of the data point is multiplied by the respective weight. To obtain the smoothed value for the central point of the window, the sum of the weighted data is divided by the sum of the weights. In addition to the window size, the standard deviation *σ* of the Gaussian function is a freely selectable parameter.

The local linear/quadratic regression (lowess/loess) smooths values by fitting a linear or quadratic function to the data points within a window using weighted least squares. Tricubic weighting is typically used, giving more weight to the nearest and less weight to the furthest points. The robust variant is more resistant to outliers but more computationally expensive as the regression is adjusted not just by simple, but by iterated weighted least squares (Cleveland, 1979).

The Savitzky-Golay filter fits a polynomial of degree *n* to the data points within a window. The window size must be at least *n* + 1 points, and it is typically centered on the point being analyzed. The result of the filter is a smoothed value for the center point within the window (Savitzky and Golay, 1964). The degree *n* of the polynomial function is a freely selectable parameter.

In addition to the variance inhomogeneity of the signal over time, the change of the signal mean is another factor of non-stationarity. To address this issue, it is advisable to transform the signal in such a way that the mean of the transformed signal remains constant over time. To achieve this, a double rolling aggregate (DRA) can be used. This transformer consists of two windows, which can be freely sized, moving in parallel along the time axis over the data series. These windows can move side by side or overlap, and within each window, the data are aggregated according to the chosen aggregation function. The DRA compares the aggregated metrics of the two windows by subtracting the metric of one window from the metric of the other and saves those differences as the transformed signal. So if there is a sudden increase in signal, it is first reflected in the metric of the right window. Consequently, the difference between both window metrics, i.e., the transformed signal, also increases significantly.

### 2.2 Anomaly detection

To assess whether the difference between the two metrics of the rolling windows, referred to as transformed signal, is significant enough to be considered an anomaly, a threshold value is required above which the corresponding signal is classified as an anomaly. However, this threshold must be chosen wisely to avoid classifying too many values as false positives if the threshold is too low, and to ensure that anomalies are still recognized as such if the threshold is too high. When choosing an appropriate threshold, there are generally two different approaches. Either a threshold is set manually based on experience and visual assessment of the transformed signal, or the threshold is set based on the location and scale estimators of the respective data. The latter approach can be applied both off-line to the entire dataset and in process simulations, when only the past and present data is available at any given time point, to the local areas defined by a predefined window. When implemented in process simulations, unlike the manual method, the threshold is not static but dynamic, adapting to the continuously provided new data. For this dynamic determination of the threshold value, various approaches are available (Jones, 2019; Berger and Kiefer, 2021), which can be expressed in the form of Equation 1. The following sections will detail three methods applied in this study.

threshold=location estimator±threshold factor*scale estimator (1)

The probably best-known and most frequently used method is the 3-sigma rule (Pearson, 2001; 2002; Chiang et al., 2003; Lin et al., 2007; Zhu et al., 2018; Jones, 2019). The 3-sigma rule states that, in a normal distribution, approximately 99.73% of the data points will fall within three standard deviations of the mean (Zhao et al., 2013). This means that the probability of a data point lying outside of this range is very low, making it a useful rule of thumb for identifying outliers. It is important to note that the anomaly detection result depends on the relationship between the threshold factor and the window size (Shiffler, 1988). For example, Berger et al. describe that when using the 3-sigma rule, the sample size must be > 10 in order to possibly detect any outliers (Berger and Kiefer, 2021). In general, the maximum threshold factor can be calculated by $\left(w-1\right)/\sqrt{w}$ with the window size *w*. As the name implies, in this method the threshold factor is set to 3, and the standard deviation from the mean (location estimator) is used as the scale estimator (Equation 2).

$$threshold=\bar{x}\pm 3*\sigma$$ (2)

However, since both the mean and especially the standard deviation are very outlier-sensitive and can be overestimated by outliers, the masking effect occurs, leading to false negatives. In addition, the 3-sigma rule assumes symmetry, which can lead to false positives if this assumption is violated (Jones, 2019). Therefore, it is advisable to use more robust methods, where the mean and standard deviation are replaced by more robust location and scale estimators. The mean can be replaced by the median, and there are two options for replacing the standard deviation. If the standard deviation is replaced by the median of absolute deviation (MAD) scale estimate, the resulting method is called the Hampel identifier (Pearson, 2005). The drawback of this more robust method is that more values tend to be identified as false positives, known as swamping (Davies and Gather, 1993; Pearson, 2005), which is the opposite of the masking effect. The MAD scale estimate is the product of the constant *b* and the MAD. The value of the constant *b* depends on the underlying distribution and can be calculated as the reciprocal value of the 75th percentile (Huber, 2011; Leys et al., 2013). For a normal distribution, *b* is 1.4826 (Davies and Gather, 1993; Rousseeuw and Croux, 1993; Chiang et al., 2003; Pearson, 2005; Lin et al., 2007). The threshold factor is usually set at 2.0, 2.5 or 3.0 (Miller, 1991). The general notation is shown in Equation 3.

$$threshold=\tilde{x}\pm threshold\;factor\;*\left(b\times median\left\{\left|x_{i}-\tilde{x}\right|\right\}\right)$$ (3)

Within a signal window, where the signal values change mainly due to the noise and not due to a process trend, normal distribution is most probable.

The other option for replacing the standard deviation with a more robust scale estimator is the interquartile range (IQR) scale estimate. It is based on the range between the 75th percentile (*Q*_*3*_) and the 25th percentile (*Q*_*1*_) and is less sensitive to outliers than the standard deviation but more sensitive than the MAD scale estimate. Similar to the MAD scale estimate, a correction factor is introduced for the IQR scale estimate, which is 1.35 for a threshold factor of 2 (Equation 4).

$$threshold=\tilde{x}\pm threshold\;factor\;*1.35*\left(\frac{Q_{3}-Q_{1}}{1.35}\right)=\tilde{x}\pm threshold\;factor\;*1.35*\sigma$$ (4)

These outlier detection limits correspond to approximately ± 2.7*σ, as the IQR divided by this correction factor leads to an unbiased estimate of the standard deviation σ for normally distributed data (Venables and Ripley, 2002; Pearson, 2005; Higgins and Green, 2008).

While there are more advanced methods for anomaly detection, such as machine learning-based and deep learning-based approaches (e.g., autoencoders), these techniques fall outside the scope of this study. The primary reason is their complexity and the requirement for sufficiently large datasets to ensure good model performance (Darban et al., 2024; Iqbal and Amin, 2024). In the context of bioprocesses, obtaining such large datasets is often challenging, as data collection is typically expensive and time-consuming. Moreover, the effective implementation of these advanced methods demands interdisciplinary expertise in data science, statistics, and bioprocess engineering, which can limit their accessibility and practical adoption in many industrial and academic settings. Therefore, we focus on threshold-based methods that are more practical given the constraints of bioprocess monitoring.

## 3 Materials and methods

Since this study refers to the data from Grigs et al., only the key aspects of the cultivation and data acquisition are described below. For details, we refer to the original study where the experimental data were recorded (Grigs et al., 2021a). HBcAg (Mut^+^) and HBsAg (Mut^S^) recombinant *P. pastoris* GS115 strains (obtained from Latvian Biomedical research and study centre) were cultivated in 5 L fully automated bench-top bioreactor systems EDF-5.4 (Biotehniskais Centrs, Riga, Latvia). Residual methanol levels varied between 0.01–7 g/L during the protein production phase, process temperature was 30°C ± 0.1 C (or 24°C ± 0.1 °C for Exp. 2) and the aeration rate was set at 3.0 slpm. The dissolved oxygen level varied between 3%–40% and the set-point of 30% ± 5% was controlled by automatically adjusting the stirrer rotational speed (200–1000 rpm) or additional inlet air enrichment with oxygen. The permittivity signal (Hamilton, Bonaduz, Switzerland, Incyte) was recorded every 60 s. According to the manufacturer, the permittivity probe has an accuracy of ± 1 pF/cm or ± 1%, whichever is greater across the full measurement range. Zero calibration was conducted before inoculation using cell-free culture media under process conditions. The duration of the time series and the scale of permittivity values for each experiment are summarized in Supplementary Table S1. Out of a dataset of 13 experiments, only eight contained permittivity sensor data and were selected for this study. The names of the eight experiments considered from the original study (1s–4s; 3c–6c) correspond to experiment numbers 1–8 in this work. The suffixes s and c in the original study refer to the particular *P. pastoris* producer strain employed, with s denoting the hepatitis B surface antigen (HBsAg) producer and c the hepatitis B core antigen (HBcAg) producer (Grigs et al., 2021b; Bolmanis et al., 2022).

MATLAB version R2021b (Mathworks, Natick, MA, USA) with the Statistics and Machine Learning Toolbox was used for algorithm code and figure creation. The algorithm was visualized using draw.io (https://drawio.com).

Algorithm implementation and calculations were performed on a desktop computer with an Intel i5-6600 (3.90 GHz) processor and 16 GB RAM.

In the development of the algorithm, we followed three steps: signal preprocessing, anomaly detection, and anomaly validation and removal. Accordingly, this chapter will go through these steps in sequence.

### 3.1 Signal preprocessing

To evaluate the performance of the smoothing method, a reference signal with no noise is required. The permittivity signals were first smoothed off-line, utilizing the complete dataset to obtain a ‘noiseless’ signal as a reference. Several methods, namely, the moving mean and median, Gaussian filter, Savitzky-Golay filter, local linear and quadratic regression, and their robust equivalents were applied and the results were visually compared in terms of preserving the original signal pattern and removing most signal fluctuations.

Once the optimal off-line data smoothing method was identified, the resulting ‘noiseless’ signal served as a reference. The noise in the smoothed signals was then assessed by calculating the average normalized root mean square error (NRMSE) for each experimental dataset (Equation 5).

$$NRMSE=\frac{\sqrt{\frac{\sum_{i=1}^{n}{\left(y_{i}-{y_{i}}^{*}\right)}^{2}}{n}}}{y_{\max}-y_{\min}}100\%$$ (5)

Where *y*_*i*_ is the *i*th reference noiseless permittivity signal value, *y*_i_^*^ is the smoothed permittivity signal value, *y*_min_ and *y*_max_ are the minimum and maximum values of reference *y*_*i*_.

Higher signal fluctuations (noise) directly correspond to an increased NRMSE value. In contrast to the previous step, this smoothing was performed in fermentation process simulations, where only past and present (not future) data is available to the model at any given time. Several different signal filtration methods and parameters were investigated using the *smoothdata* function (MATLAB). Namely, moving mean and median, Gaussian filter, Savitzky-Golay filter, local linear and quadratic regression, and their robust equivalents. For each of these methods, the optimal smoothing window sizes were identified, achieving the best signal noise reduction performance, as indicated by the lowest NRMSE. Since signal filtration in real-time often introduces a signal delay, this delay must also be taken into account. The smoothed signal delays in process simulations were estimated using signal cross-correlation function *xcorr* (MATLAB) comparing the transformed raw and smoothed permittivity signals. Cross-correlation is a widely used technique for estimating signal delay by measuring the similarity between two signals as a function of time lag. In this approach, one signal is systematically shifted relative to the other, and their correlation is computed at each shift. The time lag corresponding to the maximum correlation value indicates the estimated delay between the signals (Müller et al., 2003).

For all methods, we investigated window sizes of 2–180 data points, corresponding to 2–180 min. For the Gaussian filter, the standard deviation was fixed to be 1/5th of the total window width. A lowess/loess smoothing technique was applied using weighted linear or quadratic least squares with a first- or second-degree polynomial model. The degree *n* of the polynomial function for the Savitzky-Golay filter was set to two.

For the transformation of the smoothed signal to remove context dependency, we used the DRA with the mean as aggregate function. The window sizes *w1* and *w2* varied from one to 20 with increments of one; the windows did overlap entirely.

### 3.2 Anomaly detection

The anomaly detection is based on the selection of an appropriate threshold, based on which an anomaly is classified as such. For this, the approaches outlined in the theory section were compared. On the one hand, this includes the manual method, where the optimal threshold value was determined from a range from 0.10 to 1.45 in increments of 0.01. The other approaches are based on the real-time implementation of a dynamic threshold. In addition to the 3-sigma rule, we applied the Hampel identifier where we set the factor *b* to 1.4826, since we assume normal distribution. In accordance with the 3-sigma rule, the threshold factor was set to three. Furthermore, we exerted the threshold determination using the IQR scale estimate with ± 2.7*σ. The performance of the method was compared as described below.

First, the signal anomalies for each experiment were manually annotated (Supplementary Table S1). The decrease in the permittivity signal after approx. 20–30 h was not considered anomalous as it was due to switching the feed substrate from glycerol to methanol, which is usually followed by an adaptation period corresponding to a reduced cell viability and little to no growth for approx. 1–2 h (Ferreira et al., 2014). Then, the threshold methods were applied to the transformed signals of each experiment. Various window *w3* sizes were chosen between 120 and 180 for the 3-sigma method and from two to 30 for the MAD and IQR method. In both cases, *w3* was varied in increments of two. To determine which window size produced the best results, the true positives (TP), false positives (FP) and false negatives (FN) were analyzed by comparing the detected anomalies with the annotated anomalies. Using these measures, the precision (TP/(TP + FP)), recall (TP/(TP + FN)) and F1-score (2 * precision * recall/(precision + recall)) were calculated. The best results are indicated by the highest average F1-score across all experiments. In this way, the window size for each method that achieves the best results (i.e., the highest average F1-scores) can be determined. Finally, the highest F1-scores of the different methods can be compared to identify the best method. Exp. 6 was omitted from this calculation as no significant signal anomalies were detected for this experiment.

### 3.3 Anomaly removal

If a signal anomaly is detected, the permittivity signal will be corrected by replacing the anomalous value with the mean of the previous 15 values. Once the anomaly has passed, a 15-min validation window begins to estimate the new signal baseline. The baseline level before the anomaly is then subtracted from the baseline level after the anomaly to determine a correction term, which is applied to the permittivity signal.

## 4 Results and discussion

To address the in-line permittivity sensor signal anomalies during recombinant yeast *P. pastoris* fermentations, we developed an algorithm for real-time detection and removal of these anomalies. The algorithm consists of three consecutive steps: 1) signal preprocessing, 2) anomaly detection, 3) anomaly validation and removal. Each step is detailed in the following sections.

### 4.1 Signal preprocessing

By analyzing the experimental dataset, we found that the permittivity signal contains significant noise and the overall signal quality should be subject to improvement. The signal noise was also even more prominent in two experiments (3 and 4), which were conducted in a different cultivation medium, indicating that the permittivity signal noise could be affected, for example, by medium conductivity. Henceforth, we found it essential to include a prerequisite signal noise filtering step to improve overall signal quality prior to anomaly detection.

**FIGURE 1:**
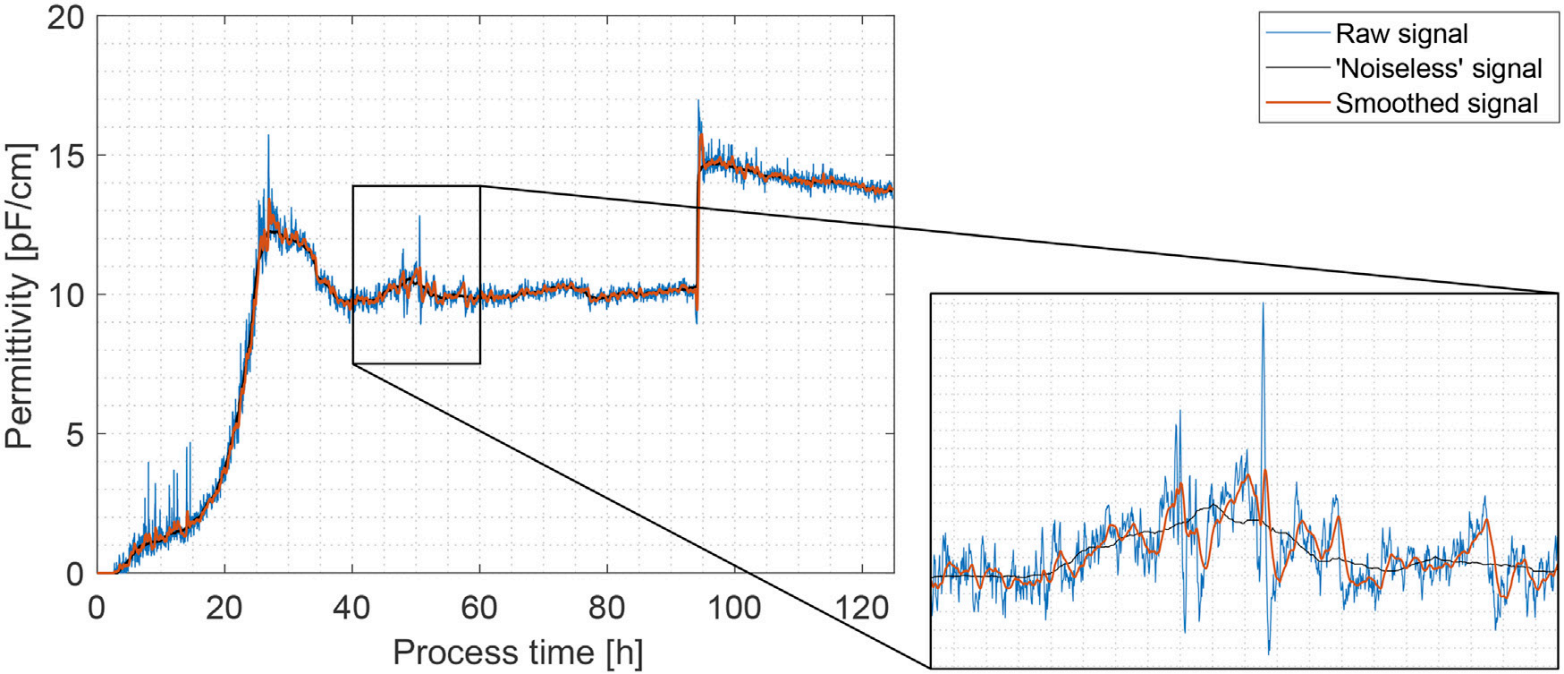
Comparison of raw (colored in blue), ‘noiseless’ (off-line ‘loess’ filter) (colored in black) and real-time smoothed (Gaussian filter, *w* = 70) (colored in orange) permittivity signals in Exp. 1 for signal noise filtering method performance estimation.

‘Noiseless’ reference signals were obtained by smoothing the raw permittivity signals off-line utilizing the whole dataset with different methods. The best results with high preservation of the original signal pattern and removing most signal fluctuations were achieved by using a local quadratic regression smoothing filter with a smoothing factor of 0.03 (see Figure 1). Other smoothing methods failed to fully encapsulate the underlying signal characteristics by either cutting off distinctive signal peaks or misrepresenting anomalous signal jumps and spikes.

The smoothing performance of various methods was evaluated by calculating the NRMSE between the ‘noiseless’ reference and the smoothed signals in fermentation process simulations utilizing only past and present data at any given time point, as well as estimating the signal delay between the raw and smoothed signals. The best results were achieved, using a Gaussian smoothing filter with a window size of 70. In this case, an average NRMSE of 4.56% with a standard deviation of ± 1.40% was achieved (in comparison to 6.76% ± 1.93% for the raw signal) with an average estimated signal delay over all experiments of 6.4 min. Similar performance was noted by the moving mean filter (4.89% ± 1.55%), however, significantly higher signal delays of an average of 10.1 min were noted. The performance of other methods were deemed unsatisfactory either due to higher NRMSE values or prolonged signal delays. In the case of robust local linear/quadratic regressions (rloess/rlowess), a significantly higher computational burden was noted and thus these methods were excluded from consideration for real-time signal smoothing implementation. For the extended results, we refer to Supplementary Table S2.

As a result of this step, a higher quality permittivity signal was produced for signal anomaly detection in the next step. Much of the signal noise was removed and, although slight signal delays were introduced (which is to be expected), we estimate that they are not significant enough not to warrant using the filtered signal for real-time substrate feed rate adjustment in yeast or mammalian cell fed-batch bioprocesses, for example. Of course, the acceptable signal delay is highly process-specific and should be considered with every application. The average specific growth rate for *P. pastoris* Mut^+^ phenotype on methanol varies between 0.02 and 0.15 h^-1^ (Looser et al., 2015). This represents an average biomass increase by 2.0%–15.0% every hour. Hence, a signal delay of 5–10 min can be considered insignificant. The results for signal real-time smoothing in Exp. 1, using a Gaussian filter with a window size of 70, are shown in Figure 1. Permittivity signal preprocessing is used quite often when employing an in-line sensor probe (Ramm et al., 2023), however, necessary signal quality is often determined by the way the signal is to be used. For example, some authors have used the permittivity signal only for monitoring purposes, thus choosing not to apply any additional signal processing steps (Sarrafzadeh et al., 2005; Meitz et al., 2016; Pentjuss et al., 2023; Sakiyo and Németh, 2023). On the other hand, when choosing (or by necessity) to filter the permittivity signal, a moving average filter or a variation of it is often employed with window sizes varying from 15–110 samples (Da Silva et al., 2013; Downey et al., 2014; Horta et al., 2015). Horta et al. thereby developed a smoothed moving average filter, which performed better in permittivity sensor signal noise reduction than a classical moving average filter (Horta et al., 2012). The filtered signal was then used to estimate the cell growth rate (μ) and control the substrate feed rate in *E. coli* cultivations. The authors also emphasize that an efficient noise filter was essential for a good performance of the control system. A similar control strategy was also employed by Da Silva et al. (2013).

### 4.2 Anomaly detection

**FIGURE 2:**
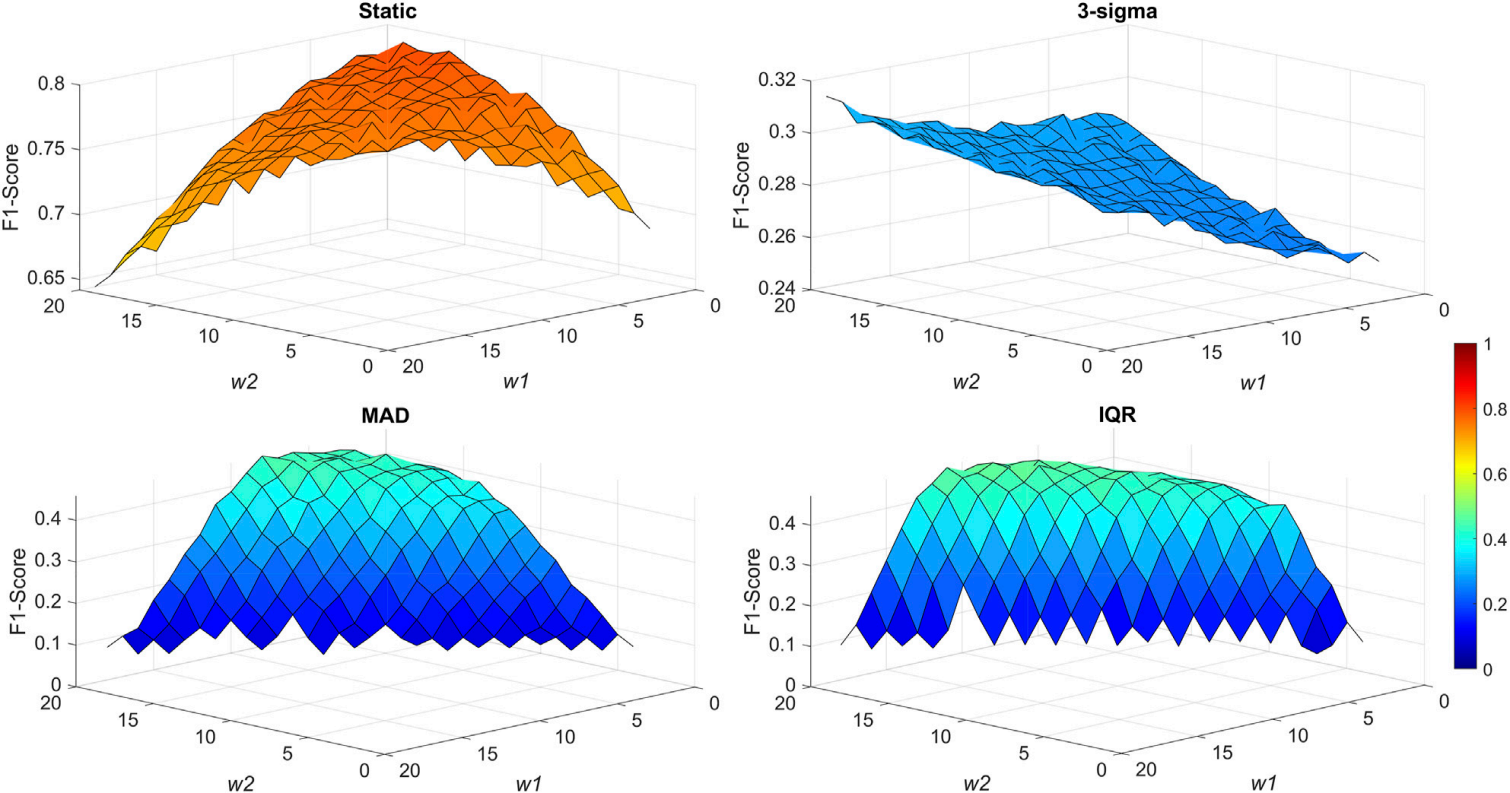
Highest F-scores achieved for respective types of thresholds at selected DRA transformer window sizes *w1* and *w2* with optimized threshold values (for static threshold type) and window sizes *w3* (for dynamic threshold types).

For signal anomaly detection using the DRA transformer, we investigated four different strategies for anomaly threshold determination. In addition to the manually selected static threshold, we tested three different variants of a dynamic threshold based on the 3-sigma rule, MAD and IQR scale estimates. The respective resulting F1-scores are shown in Figure 2. We also examined consecutive (once per selected period, *w4*) dynamic threshold calculations, however, in all cases, a lower F-score value was achieved and, thus, this method was discarded.

As can be seen in Figure 2, the best anomaly detection performance was demonstrated using the static threshold. An F1-score of 0.7935 was achieved with window sizes of *w1* = 1, *w2* = 15, and a threshold value of 1.06 pF/cm. An F1-score of 0.8 is usually considered a good result (Fränti and Mariescu-Istodor, 2023) as the algorithm demonstrates good anomaly prediction performance. The static threshold method was also the least computationally expensive and easy to implement, in comparison to the dynamic threshold methods, thus promoting its use in real-time process implementation.

The dynamic threshold methods produced significantly lower F1-scores, all of which were below 0.5 and can be considered as not good enough. The MAD and IQR approaches produced similar results, as the methods are quite similar themselves. In both cases, the F1-score was significantly impacted by the detection of false positive and false negative anomalies. Regarding the 3-sigma threshold, the performance was similar to the static threshold in detecting true positive and false negative anomalies, however, a very high number of false positive anomalies were detected, impacting the overall F1-score. For extended results, refer to Supplementary Table S3.

**FIGURE 3:**
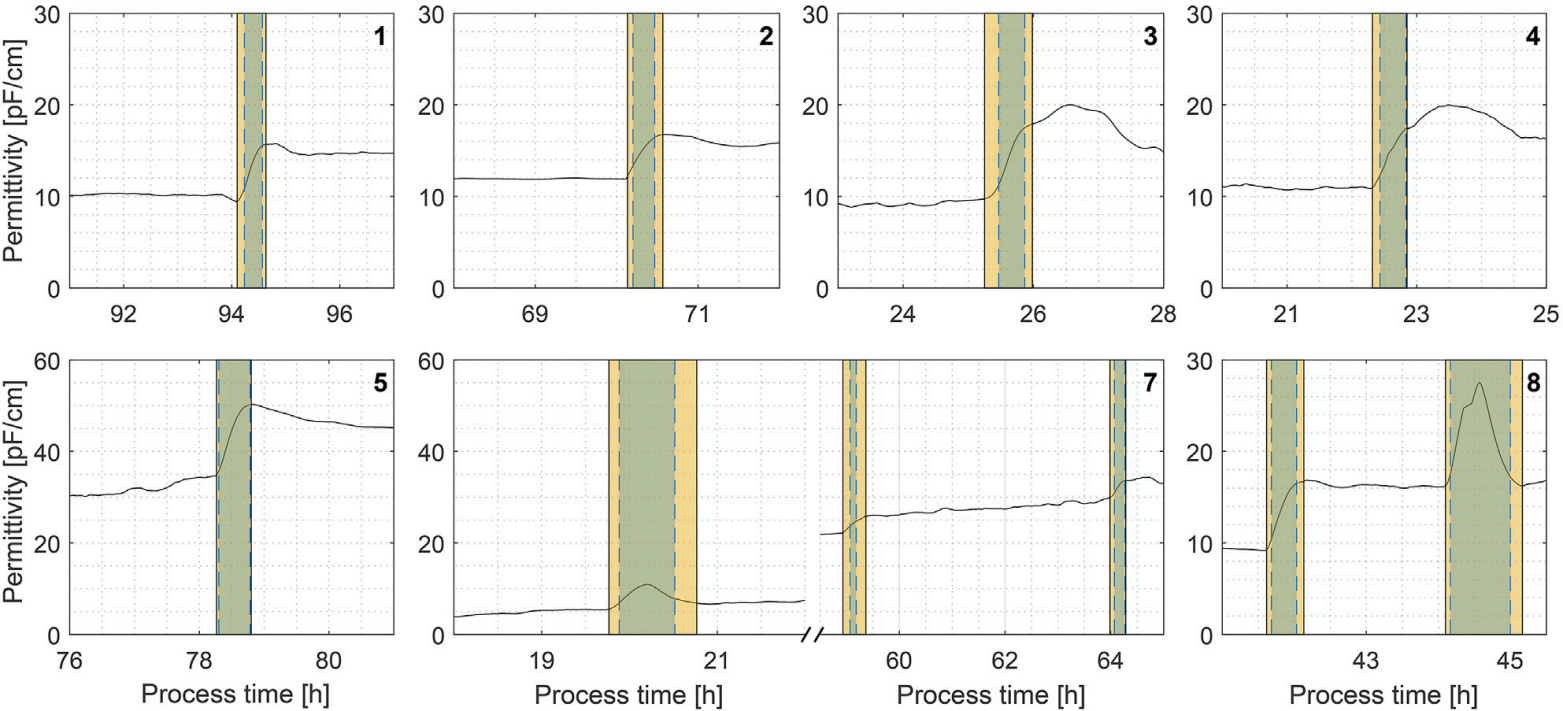
Comparison of manually annotated permittivity signal anomalies (orange shading) vs. successfully detected anomalies (blue shading) in fermentation process simulations. Exp. 6 was omitted because it contained no detected anomalies.

With the static threshold approach, the F1-score criterion, referred to as ‘precision’, was 1.0 across all processes, indicating that every anomaly detected was in fact an anomaly. The other criterion, ‘recall’, demonstrating how many of all signal anomalies were correctly identified, varied from 0.47 to 0.91. On average, 20 anomalous signal data points were not detected in each process (false negatives). Although that may seem significant initially, this count mainly arises from undetected anomalous signal values just prior and after detected signal anomalies (see Figure 3). The orange shading indicates manual anomaly annotations, and the blue shading shows the algorithm-detected anomalies. Overlap indicates good performance (e.g., Exp. 5). Most false negatives in the F-test result from slight delays in detection or early cutoffs as the signal flattens after an anomaly. It is in part caused by signal smoothing, as the signal change before and after anomalies is not so sudden and prominent anymore, hence the spike in the DRA transformed signal is also slightly delayed. In this case, it can be envisioned as a tradeoff between signal anomaly detection time and overall detection robustness.

Additionally, it can be noted that in most cases (excluding the 3-sigma threshold), *w1* size was quite low (1 or 2). This corresponds to the swiftness of anomaly detection, as with a smaller *w1* size, the anomalies are detected more quickly due to the mean of the window increasing more rapidly due to sudden signal jumps. With greater window sizes, the increase is slower, however, the detection can be seen as more robust.

In the case with all of the dynamic thresholds, the results were worse than expected. The dynamic threshold calculations are carried out, based on past signal values, hence, if the signal volatility suddenly increases, the dynamic threshold value increase is delayed by design. For example, if signal volatility has been low, the dynamic threshold is also low, but, if the volatility suddenly increases, the signal threshold is still low, thus, signal anomalies are detected. Assuredly, this may not be a problem when implementing such algorithms off-line (using the whole dataset), but in a real-time implementation this phenomenon could only be overcome by introducing some sort of signal volatility prediction parameter, which is beyond the scope of this article.

### 4.3 Anomaly validation and removal

In the final step, the detected permittivity signal anomalies are removed by introducing an alternative (corrected) permittivity sensor signal. When an anomaly is detected, the signal is corrected by replacing the current permittivity value with a mean of 15 past values prior to anomaly detection. Thus, the sudden nature of signal anomalies does not interfere, for example, with substrate feed rate calculations. On the other hand, the sudden increase in permittivity signal value would be estimated as a sudden increase in viable cell concentration by the substrate feeding algorithm and, thus, a drastic corrective action of the feed rate would follow. Such severe alterations to the substrate feeding profile would certainly lead to profound negative effects on process productivity and even result in batch discard.

This phase is initiated just after the detection of a signal anomaly and is characterized by an anomaly validation period of 15 min. During this transition period, the permittivity signal correction continues, estimating the corrected signal as a mean of past 15 values. In the case of a signal spike, anomaly detection is triggered by a sudden signal jump upwards. However, it is always followed by a sudden signal drop of similar magnitude. In such cases, the second anomaly often falls within the validation period. If so, then both anomalies are grouped into one and, due to the nature of these signal spikes, the corrective action is often minor as, after the anomaly, the signal returns to its previous level.

**FIGURE 4:**
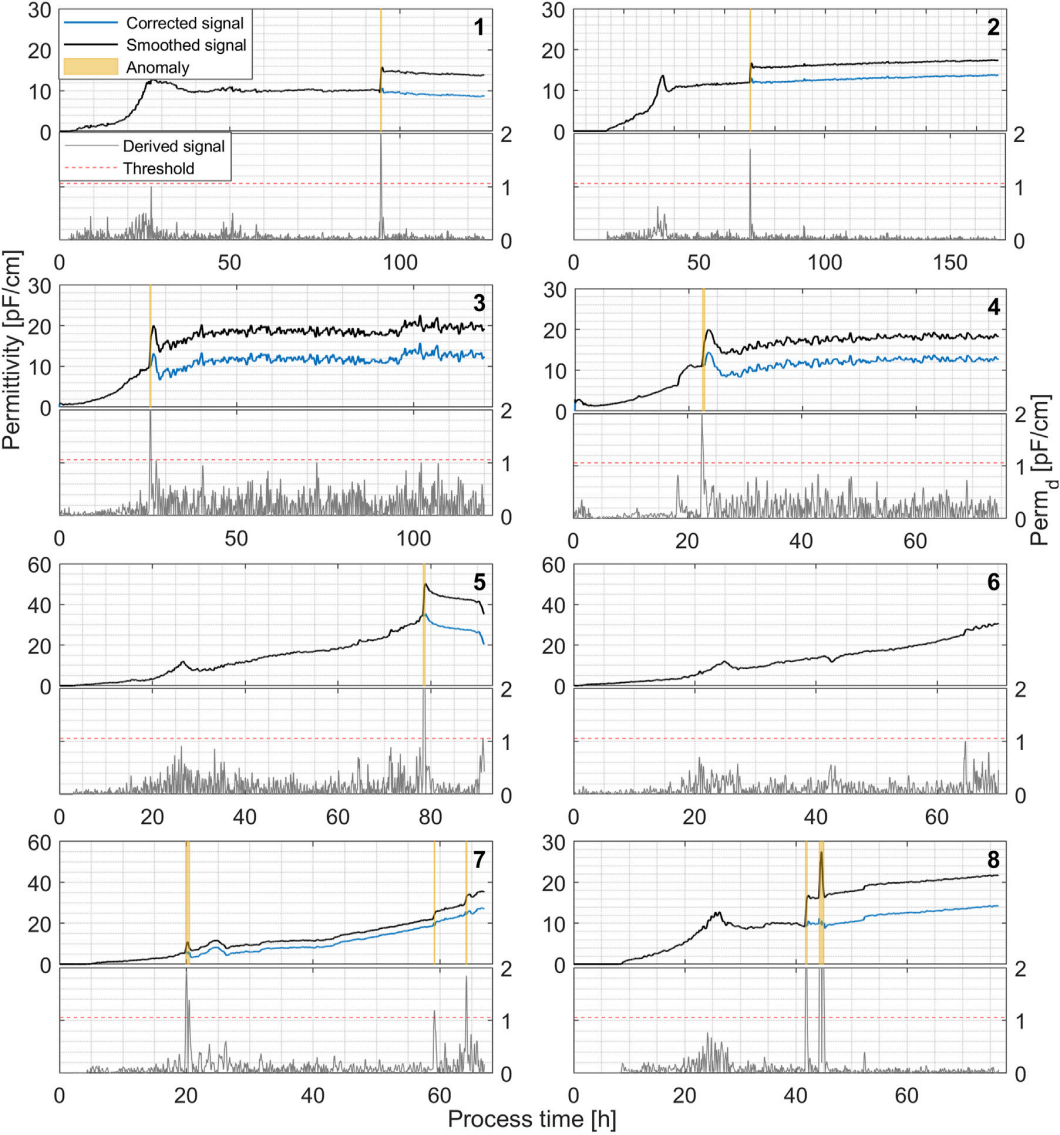
Permittivity signal anomaly detection and removal algorithm performance in real-time simulated recombinant *P. pastoris* fermentation processes. Raw signal is filtered in real-time using a Gaussian smoothing filter (*w* = 70) and a DRA-transformed (lower plot) Perm_d_ signal (*w1* = 1, *w2* = 15) is used for anomaly detection with a static threshold of 1.06 pF/cm.

If a signal shift occurs, it is detected as a single signal anomaly. In this case, during the validation period, the new signal level is estimated. If additional anomalies are detected within the initial 15-point window, the window is dynamically extended to ensure that at least 15 min of valid data follow the last detected anomaly. A correction factor (Fc) is then introduced to compensate for the signal shift that has occurred. Fc is estimated as the difference between 15 mean signal values before and after (validation period) anomaly detection. This value is then used to compensate for the previous signal values and introduce the corrected signal (see Figure 4).

### 4.4 Anomaly detection and removal algorithm

**FIGURE 5:**
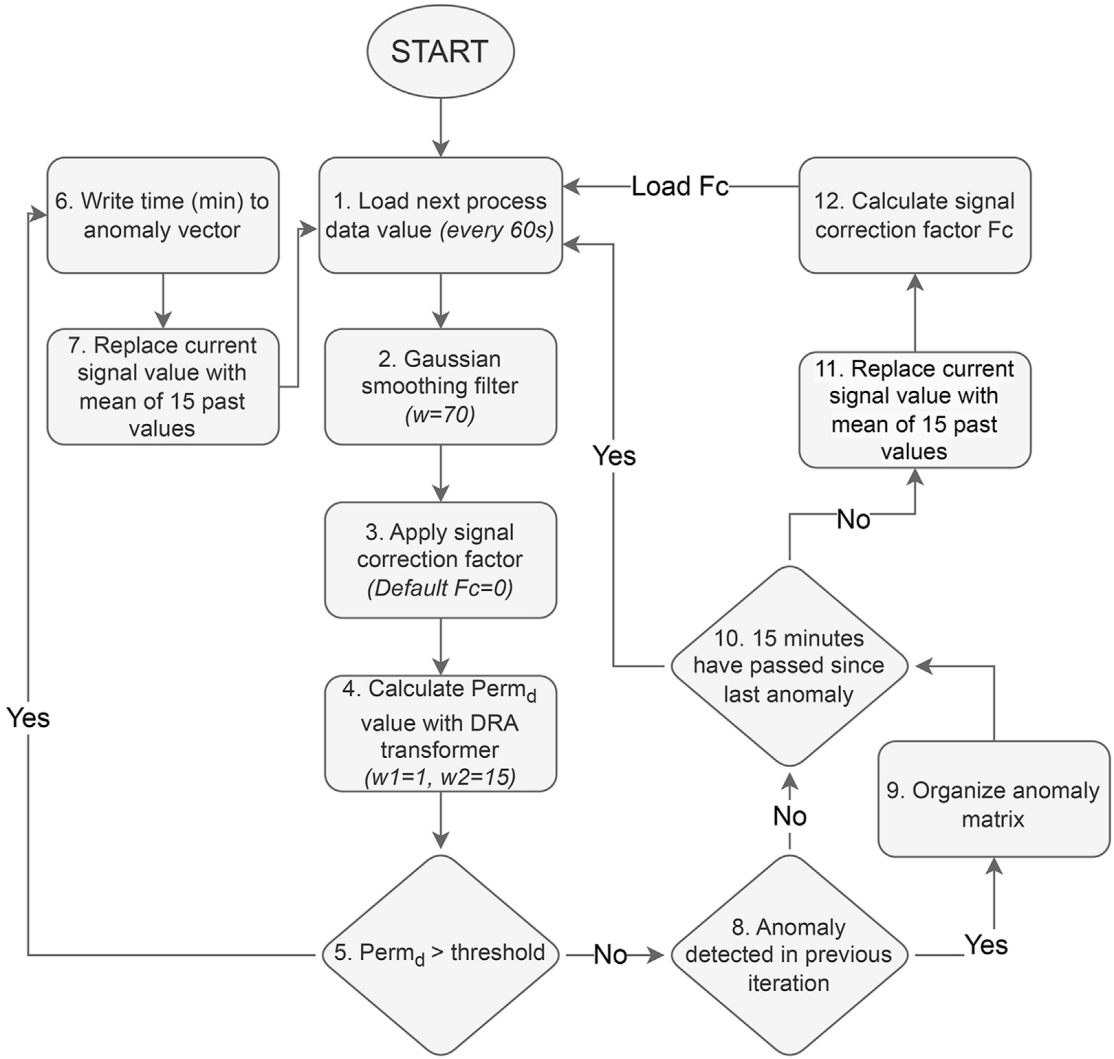
Schematic representation of the implemented permittivity signal anomaly detection and removal algorithm.

The combination of the aforementioned steps resulted in the creation of a novel permittivity signal anomaly removal algorithm. The algorithm is implemented in real-time simulations of recombinant *P. pastoris* fed-batch bioprocesses and effectively detects and removes in-line permittivity sensor signal anomalies. The schematic representation of the algorithm can be seen in Figure 5.

The bioreactor data processing program operates in a loop, loading data every minute (Step 1). The program then preprocesses the signal using a Gaussian smoothing filter and applies the signal correction factor Fc (initially set to zero) (Steps 2–3). A derived signal value (Perm_d_) is then calculated using the DRA transformer (*w1* = 1, *w2* = 15) and compared to a threshold of 1.06 pF/cm (Step 5). If Perm_d_ exceeds the threshold, the time point is logged as an anomaly (Step 6), and the signal value is replaced by the mean of the past 15 values (Step 7) and the program returns to Step 1.

If the Perm_d_ value does not exceed the threshold, then the algorithm evaluates whether an anomaly was previously detected in the previous iteration (Step 8). In all cases, the anomalies were registered as strings of consecutive time series, thus, if an anomaly was detected in the previous iteration and is not detected anymore in the next step, it indicates that the anomaly has passed. Furthermore, the anomaly can now be organized into the anomaly matrix, registering the anomaly start time in column 1 and end time in column 2 (Step 9).

Signal spikes are often detected as two separate anomalies, as both the initial signal jump and subsequent drop are detected by the DRA transformer. To avoid unnecessary signal overcorrection, a 15-min anomaly validation period was implemented (Step 10). Multiple anomalies within these 15 min are merged together as a single anomaly mainly to filter out signal spikes. When a signal spike occurs, the signal tends to return to the previous level after the spike has passed, thus, minimal or no corrective action is usually necessary.

During the validation period, permittivity signal values are replaced with means of 15 past values to compensate for the signal level after the shift, and a signal correction factor Fc is calculated from signal values before and after the anomaly (Steps 11–12). The anomaly validation period is also crucial for Fc calculation as the 15-min window provides a chance to estimate the extent of the permittivity signal shift. The correction factor Fc is calculated by subtracting the mean of 15 permittivity signal values prior to a detected anomaly from the mean of 15 values after an anomaly. It is then used in subsequent iterations to compensate for the permittivity signal shift that occurred during each detected signal anomaly.

The particular algorithm, when implemented in MATLAB, managed to successfully detect and remove permittivity signal anomalies for the selected dataset in real-time process simulations with an average computation time per iteration loop of 0.32 milliseconds, greatly improving overall permittivity signal quality. Thus, proving to be a rather straightforward and easy to implement tool for real-time permittivity signal anomaly removal, promoting the use of viable cell concentration measurement for substrate feed rate calculation in fed-batch bioprocesses.

The exact cause of these permittivity signal anomalies remains unclear, however, a significant correlation can be established with antifoam solution addition and changes in agitation, which often precede said anomalies. Studies have demonstrated that introducing small quantities of antifoam leads to a reduction in gas hold-up and an increase in average bubble diameter. This enlargement of bubble size results in a decreased specific surface area and medium surface tension (Al-Masry et al., 2006; Routledge, 2012). We presume that antifoam addition increases culture medium density primarily by reducing entrapped air bubbles, facilitating the formation of larger bubbles that rise and escape more easily, thereby decreasing gas hold-up. This reduction in gas volume results in a denser liquid phase, which accounts for the consistent upward shifts in permittivity signals. Previous studies have reported that antifoam addition correlates with increased culture density (Routledge et al., 2011). This theory is also supported by visual assessment of *P. pastoris* cultivation media volume prior and after antifoam addition. This suggests that incorporating small amounts of antifoam at the start of fermentation could be beneficial, provided it is compatible with the selected microorganism and bioprocess.

## 5 Conclusion

This study tackles a key challenge in *in-situ* measurement related to viable biomass concentration: the development of a robust and easily implementable algorithm for real-time anomaly detection and removal in permittivity sensor data. Unlike simplistic methods, which fail to capture the complexity of the issue, our approach offers a structured three-step solution: (1) Signal preprocessing to minimize noise and remove context dependency; (2) Anomaly detection through threshold-based identification; and (3) Validation and removal of detected anomalies.

As a result, we present a general workflow with defined steps for in-line permittivity sensor signal anomaly detection and removal. This approach enabled reliable real-time anomaly detection and removal in permittivity sensor data from recombinant *P. pastoris* fermentations while maintaining computational efficiency, making it practical for real-time applications. With a static threshold of 1.06 and a double rolling aggregate transformer using window sizes *w1* = 1 and *w2* = 15, it achieves an F1-score of 0.79. This flexible algorithm has the potential to bridge a critical gap in process analytics and control for real-time bioprocess monitoring, while its ease of implementation promotes the use of in-line permittivity measurements in monitoring and control applications in other cultivations.

## Data Availability

Online records of bioreactor parameters and raw in-line permittivity sensor data analyzed for this study can be found under https://dx.doi.org/10.5281/zenodo.14264619.
