# Supplementary material for: Anomaly detection and removal strategies for in-line permittivity sensor signal used in bioprocesses
Source: Front Bioeng Biotechnol. 2025 Jul 30;13:1609369. doi: 10.3389/fbioe.2025.1609369 (PMC12343699; doi:10.3389/fbioe.2025.1609369)
Supplement: Supplementary file 1 [file Table1.docx]

Supplementary Material

# Supplementary Tables

Table S1. Manually annotated anomalies in and the characteristics of the experimental dataset.

| **Exp No.** | **Anomaly timestamp (process min.)** | **Detected anomaly type** | **Time series length (min.)** | **Max permittivity (pF/cm)** |
| --- | --- | --- | --- | --- |
| **1** | 5646-5678 | Shift | 7484 | 16.97 |
| **2** | 4208-4234 | Shift | 10122 | 17.49 |
| **3** | 1515-1559 | Shift | 7199 | 24.52 |
| **4** | 1339-1371 | Shift | 4469 | 21.67 |
| **5** | 4696-4728 | Shift | 5489 | 51.75 |
| **6** | - | - | 4205 | 32.79 |
| **7** | 1186-1246  3536-3562  3839-3857 | Spike  Shift  Shift | 4019 | 37.12 |
| **8** | 2497-2528  2646-2710 | Shift  Spike | 4591 | 30.66 |

Table S2. NRMSEs and signal delays of various smoothing methods against reference signal.

| **Exp. No.** | **Gaussian** | | **Moving mean** | | **Moving median** | | **Loess** | | **Lowess** | | **S-Golay** | | **No smoothing** | |
| --- | --- | --- | --- | --- | --- | --- | --- | --- | --- | --- | --- | --- | --- | --- |
|  | *w* = 70 | delay (min) | *w* = 45 | delay (min) | *w* = 30 | delay (min) | *w* = 3 | delay (min) | *w* = 120 | delay (min) | *w* = 15 | delay (min) |  | delay (min) |
| **1** | 4.78% | 6 | 5.09% | 12 | 6.01% | 7 | 8.02% | 0 | 5.26% | 6 | 8.52% | 1 | 8.02% | 0 |
| **2** | 3.16% | 6 | 3.29% | 11 | 3.96% | 8 | 3.91% | 0 | 3.75% | 7 | 4.07% | 1 | 3.91% | 0 |
| **3** | 6.25% | 8 | 6.79% | 11 | 8.00% | 7 | 8.18% | 0 | 7.98% | 9 | 8.73% | 0 | 8.18% | 0 |
| **4** | 5.61% | 6 | 6.02% | 12 | 7.10% | 7 | 8.49% | 0 | 7.01% | 7 | 9.52% | 0 | 8.49% | 0 |
| **5** | 4.12% | 6 | 4.34% | 11 | 4.84% | 7 | 6.01% | 0 | 4.78% | 7 | 6.21% | 1 | 6.01% | 0 |
| **6** | 2.78% | 5 | 2.97% | 5 | 3.35% | 7 | 5.53% | 0 | 3.02% | 5 | 5.87% | 1 | 5.53% | 0 |
| **7** | 3.45% | 6 | 3.74% | 8 | 3.86% | 7 | 4.82% | 0 | 4.55% | 7 | 5.14% | 1 | 4.82% | 0 |
| **8** | 6.32% | 8 | 6.90% | 11 | 8.86% | 7 | 9.11% | 0 | 7.71% | 9 | 9.65% | 0 | 9.11% | 0 |
| **AVG:** | **4.56%** | **6.38** | **4.89%** | **10.13** | **5.75%** | **7.13** | **6.76%** | **0** | **5.51%** | **7.13** | **7.21%** | **0.63** | **6.76%** | **0.00** |

Table S3. Detailed highest F1-score results for each threshold type using the optimal parameters.

|  | **Exp.** | **TP** | **FP** | **TN** | **FN** | **Precision** | **Recall** | **F1-score** | **F1-score average** |
| --- | --- | --- | --- | --- | --- | --- | --- | --- | --- |
| **Static threshold** *w1*=1  *w2*=15  value= 1.06 | 1 | 21 | 0 | 7452 | 12 | 1.00 | 0.64 | 0.78 | **0.79** |
|  | 2 | 17 | 0 | 10096 | 10 | 1.00 | 0.63 | 0.77 |  |
|  | 3 | 25 | 0 | 7155 | 20 | 1.00 | 0.56 | 0.71 |  |
|  | 4 | 25 | 0 | 4437 | 8 | 1.00 | 0.76 | 0.86 |  |
|  | 5 | 30 | 0 | 5457 | 3 | 1.00 | 0.91 | 0.95 |  |
|  | 7 | 50 | 0 | 3913 | 57 | 1.00 | 0.47 | 0.64 |  |
|  | 8 | 70 | 0 | 4483 | 27 | 1.00 | 0.72 | 0.84 |  |
| **3-sigma dynamic threshold**  *w1*=19  *w2*=20  *w3*=120  continuous | 1 | 24 | 155 | 7297 | 9 | 0.13 | 0.73 | 0.23 | **0.31** |
|  | 2 | 21 | 158 | 9938 | 6 | 0.12 | 0.78 | 0.20 |  |
|  | 3 | 24 | 68 | 7087 | 21 | 0.26 | 0.53 | 0.35 |  |
|  | 4 | 20 | 52 | 4385 | 13 | 0.28 | 0.61 | 0.38 |  |
|  | 5 | 18 | 96 | 5361 | 15 | 0.16 | 0.55 | 0.24 |  |
|  | 7 | 48 | 88 | 3825 | 59 | 0.35 | 0.45 | 0.40 |  |
|  | 8 | 38 | 60 | 4423 | 59 | 0.39 | 0.39 | 0.39 |  |
| **MAD dynamic threshold**  *w1*=8  *w2*=20  *w3*=2  continuous | 1 | 19 | 28 | 7424 | 14 | 0.40 | 0.58 | 0.48 | **0.46** |
|  | 2 | 15 | 18 | 10078 | 12 | 0.45 | 0.56 | 0.50 |  |
|  | 3 | 24 | 95 | 7060 | 21 | 0.20 | 0.53 | 0.29 |  |
|  | 4 | 20 | 67 | 4370 | 13 | 0.23 | 0.61 | 0.33 |  |
|  | 5 | 26 | 62 | 5395 | 7 | 0.30 | 0.79 | 0.43 |  |
|  | 7 | 35 | 27 | 3886 | 72 | 0.56 | 0.33 | 0.41 |  |
|  | 8 | 68 | 14 | 4469 | 29 | 0.83 | 0.70 | 0.76 |  |
| **IQR dynamic threshold**  *w1*=8  *w2*=17  *w3*=2  continuous | 1 | 21 | 28 | 7424 | 12 | 0.43 | 0.64 | 0.51 | **0.47** |
|  | 2 | 16 | 21 | 10075 | 11 | 0.43 | 0.59 | 0.50 |  |
|  | 3 | 26 | 115 | 7040 | 19 | 0.18 | 0.58 | 0.28 |  |
|  | 4 | 23 | 72 | 4365 | 10 | 0.24 | 0.70 | 0.36 |  |
|  | 5 | 27 | 62 | 5395 | 6 | 0.30 | 0.82 | 0.44 |  |
|  | 7 | 41 | 38 | 3875 | 66 | 0.52 | 0.38 | 0.44 |  |
|  | 8 | 70 | 16 | 4467 | 27 | 0.81 | 0.72 | 0.77 |  |
